# Low Levels of Adropin Predicted New Incidents of Atrial Fibrillation in Patients with Heart Failure with Preserved Ejection Fraction

**DOI:** 10.3390/biom15081171

**Published:** 2025-08-15

**Authors:** Tetiana A. Berezina, Oleksandr O. Berezin, Evgen V. Novikov, Alexander E. Berezin

**Affiliations:** 1 Department of Internal Medicine and Nephrology, VitaCenter, 69000 Zaporozhye, Ukraine; talexberezina@gmail.com; 2 Luzerner Psychiatrie AG, 4915 St. Urban, 6110 Wolhusen, Switzerland; lunik.mender@gmail.com; 3 Department of Functional Diagnostics, Shupyk National Healthcare University of Ukraine, 04136 Kyiv, Ukraine; doctornovikov@ukr.net; 4 Department of Internal Medicine II, Division of Cardiology, Paracelsus Medical University, 5020 Salzburg, Austria

**Keywords:** heart failure with preserved ejection fraction, atrial fibrillation, adverse cardiac remodeling, adropin, natriuretic peptide, circulating biomarkers

## Abstract

Background: Atrial fibrillation (AF) is common complication of heart failure with preserved ejection fraction (HFpEF) that sufficiently intervenes in the prognosis. The aim of the study is a) to investigate the possible discriminative value of adropin for newly onset AF in patients with HFpEF without a previous history of AF and who are being treated in accordance with conventional guideline and b) to compare it with predictive potencies of conventionally used predictors. Methods: A total of 953 patients with HFpEF who had sinus rhythm on ECG were enrolled in the study. The course of the observation was 3 years. Echocardiography and assessment of conventional hematological, biochemical parameters and biomarker assay including N-terminal brain natriuretic pro-peptide (NT-proBNP), high-sensitivity cardiac troponin T, tumor necrosis factor-alpha, high-sensitivity C-reactive protein (hs-CRP), galectin-3, interleukin-6, soluble suppressor tumorigenisity-2 (sST2) and adropin, were performed at baseline. Results: Incident atrial fibrillation was found in 172 patients with HFpEF, whereas 781 had sinus rhythm. In unadjusted rough Cox regression model, age ≥ 75 years, type 2 diabetes mellitus, chronic kidney disease (CKD) stages 1–3, left atrial volume index (LAVI) ≥ 40 mL/m^2^, NT-proBNP ≥ 1440 pmol/mL, hs-CRP ≥ 5.40 mg/L, adropin ≤ 2.95 ng/mL, sST2 ≥ 15.5 ng/mL were identified as the predictors for new onset AF in HFpEF patients. After adjusting for age ≥ 75 years, a presence of type 2 diabetes mellitus and CKD stages 1–3, the levels of NT-proBNP ≥ 1440 pmol/mL and adropin ≤ 2.95 ng/mL were independent predictors of new onset AF in patients HFpEF. We also found that discriminative value of adropin was superior to NT-proBNP, while adding adropin to NT-proBNP did not improve predictive information of adropin alone. Conclusions: adropin ≤ 2.95 ng/mL presented more predictive information than NT-proBNP ≥ 1440 pmol/mL alone for new cases of AF in symptomatic patients with HFpEF, whereas the combination of both biomarkers did not improve the predictive ability of adropin alone.

## 1. Introduction

Atrial fibrillation (AF) is one of the most prevalent arrhythmias in patients with heart failure (HF) that links adverse cardiac remodeling with an increased risk of all-cause and cardiovascular (CV) death, stroke, peripheral embolism and HF decompensation [1]. The pooled prevalence of AF in the inpatients with acutely decompensated HF with reduced (HFrEF) and preserved (HFpEF) ejection fraction was 34.4% and 42.8%, respectively [2]. Along with it, AF was detected in about 20% outpatients with HFrEF and 33% those with HFpEF [2]. To note, the prevalence rate of AF in patients with HFpEF may reach 60% [3].

Moreover, AF and HF are not only coexist but also can be plausible triggers for progressing electrical and structural atrial remodeling. They may act through the overlap of several pre-disposing factors, such as conventional CV risk factors (hypertension, smoking, dyslipidemia, older age), established cardiac diseases (coronary artery disease, cardiomyopathies, valvular heart disease) and signature of comorbidities including type 2 diabetes mellitus, chronic kidney disease, overweigh/obesity [3,4]. Despite the nature of atrial remodeling in AF patients with HFpEF differs from that of HFrEF, left atrial myopathy is closely associated with cardiac fibrosis, oxidative stress, inflammation, neurohormonal activation and leads to progression of both of these conditions [4]. It has been suggested that a disproportionate prevalence of myocardial ischemia, loss of cardiac myocytes, exaggerated accumulation of the extracellular matrix in atrial tissue in HFrEF when compared with HFpEF potentiates a more rapid progression of left atrial (LA) myopathy than left ventricular (LV) dysfunction [5]. In contrast, hypertension, metabolic comorbidities (overweight/obesity, diabetes), age-associated disorders may intervene in left atrial structure and function before an occurrence of clinical signs/symptoms of HFpEF [6]. In fact, AF is considered a powerful trigger for progression of adverse cardiac remodeling and clinical manifestation of HFpEF regardless of the presentation of other risk factors and concomitant diseases. To the best of our knowledge AF and HF are not only coexist, guideline-directed medical therapy (GDMT) can prevent new cases of AF and the progression of atrial myopathy in patients with HF. However, AF can interfere with the efficacy of some components of GDMT for HFpEF [7,8,9]. As a result, identifying modifiable risk factors for AF may play a significant role in suitable preventive approach for detecting vulnerable patients with HFpEF [10].

The myocardium in HFpEF exhibits impaired mitochondrial respiratory function and increased reactive oxygen species production, both of which are hallmarks of mitochondrial dysfunction [11]. It is closely associated with exerkine, hepatokine and adipocytokine dysfunction, mitophagy abnormality, progenitor cell alteration that directly participate in dynamic nature of metabolic adaptation to disease progression [12,13,14]. Moreover, several phenotypes of all myocardial cells (cardiac myocytes, cardiac progenitor cells, endothelial cells, macrophages, fibroblasts, etc.), which are responsible for microvascular inflammation, abnormal vasoreactivity, cardiac hypertrophy and extracellular cardiac matrix accumulation/degradation, are closely regulated not only auto/paracrine factors, but also a large spectrum of cytokines and chemokines produced by skeletal myocytes, adipocytes, hepatocytes and kidney [15,16].

In this context, biomarkers, which reflect pathophysiological stages and underlying molecular pathways of AF, might help improve screening and treatment strategies. A number of clinical studies have examined the predictive role of several biomarkers, such as cardiac biomechanical stress (natriuretic peptides), inflammation (high-sensitive C-reactive protein [hs-CRP], galectin-3, interleukin-6, tumor necrosis factor-alpha), cardiac injury (cardiac troponins) and fibrosis (matrix metalloproteinases, soluble suppression tumorigenicity-2, collagen turn-over, micro-RNAs), renal function (glomeral filtration rate, cystatin-C) in predicting new case of AF, as well as of recurrence AF after ablation or electrical cardioversion [17,18,19]. Although these biomarkers have showed promising results, their discriminative values for individuals with HFpEF remains obscure [20].

Adropin is a secreted multifunctional peptide that is responsible for metabolic flexibility and optimizing substrate utilization patterns in numerous physiological and pathological conditions [21]. Adropin expression is markedly regulated by various factors, including high-fat diet, liver X receptor alpha, estrogen receptor alpha, retinoic acid receptor-related orphan nuclear receptor, regulator of reprogramming, and peroxisome proliferator-activated receptor gamma coactivator-1 expression by inhibiting sirtuin-1 and STAT3. Adropin was found to be expressed in various tissues, including the myocardium, skeletal muscles, adipose tissue, kidney, gastrointestinal tract and brain [21,22,23].

Adropin is involved in regulating glucose and lipid homeostasis, insulin sensitivity, adipogenesis, the inflammatory response, and immune reactions by modulating multiple signaling pathways (e.g., c-Jun N-terminal kinase [JNK], cAMP activated protein kinase A, activation of the glucose transporter protein and peroxisome proliferator-activated receptor gamma, Nrf2/HO-1, vascular endothelial growth factor receptor [VEGFR]-2/phosphatidylinositol 3-kinase/AKT and VEGFR/ c-Src/ERK1/2 signaling, expressing antioxidant enzymes and Bcl-2/Bax proteins). It also alleviates endoplasmic reticulum stress responses by reducing phosphorylation of the inositol trisphosphate receptor in target cells [24,25]. Moreover, adropin acting through JNK may inhibit transforming growth factor-beta (TGF-beta)-induced fibroblast activation and fibrotic tissue remodeling and promotes tissue protection [26]. Other cardiovascular effects of adropin include the regulation of arterial stiffness and vasodilation via endothelium-derived nitric oxide bioavailability, reducing cardiac hypertrophy and fibrosis, enhancing diastolic function, anti-ischemic, anti-proliferative, anti-inflammatory and anti-apoptotic effects, mediating cardiac cells survival [27,28,29,30]. On contrary, a deficiency of adropin can disrupt the function of immune cells and inflammatory pathways. It can lead to impaired regulatory capacity of the immune system and promoting systemic, microvascular and adipose tissue inflammation.

Previous clinical studies have revealed that circulating adropin levels were lower in patients with cardiovascular (atherosclerosis, acute myocardial infarction, acute and chronic HF, cardiac cachexia, stroke) and non-cardiovascular (metabolic dysfunction-associated steatotic liver disease, acute and chronic kidney diseases, overweight/obesity, type 2 diabetes mellitus) diseases [31,32,33,34,35,36]. For patients with HF regardless of its phenotypes, circulating levels of adropin were inversely correlated with HF severity [37,38]. Along with it, low levels of adropin are likely to be an independent predictor for occurrence of HF, kidney injury and diabetes mellitus, as well as to be the biomarker of poor clinical outcomes including cardiovascular death, HF-related hospitalization and diabetes-induced kidney disease [32,37,38,39,40]. However, the discriminative potency of adropin for AF in individuals with HFpEF remains unclear and requires face-to-face comparison with conventionally used and promising biomarkers, such as natriuretic peptides, cardiac troponins, soluble suppression of tumorigenicity-2 and galectin-3. The aim of the study is (a) to investigate the possible discriminative value of adropin for new onset AF in patients with HFpEF who are being treated in accordance with conventional guideline and (b) to compare it with predictive potencies of conventionally used predictors.

## 2. Materials and Methods

### 2.1. Study Population

**Figure 1:**
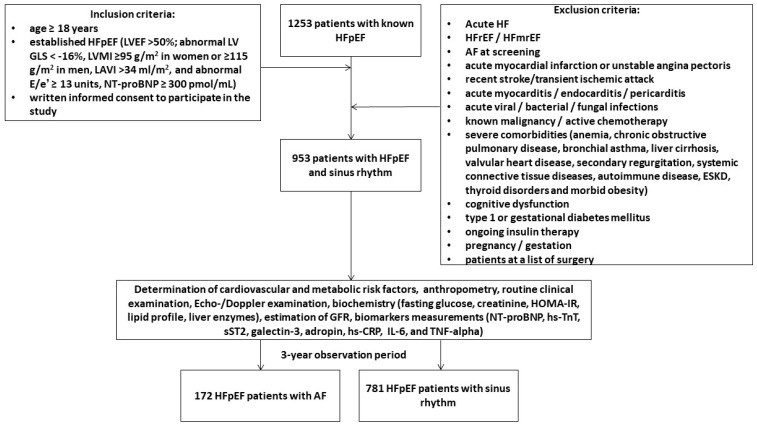
Flow chart of the study design. Abbreviations: AF, atrial fibrillation; GLS, global longitudinal stain; E/e’: the ratio of the E-wave velocity to the average of the medial and lateral e’ velocities; HF, heart failure; HFpEF, heart failure with preserved ejection fraction; HFrEF, heart failure reduced ejection fraction; HFmrEF, heart failure mildly reduced ejection fraction; HOMA-IR, the Homeostatic Assessment Model of Insulin Resistance; LVEF, left ventricular ejection fraction; LVMI, left ventricular mass index; LAVI, left atrial volume index; TNF-alpha, tumor necrosis factor-alpha; hs-CRP, high-sensitivity C-reactive protein; hs-TnT, high-sensitivity troponin T; IL, interleukin; sST2, soluble suppression of tumorigenicity-2; NT-proBNP, N-terminal natriuretic pro-peptide. Note: 300 patients were excluded in accordance with exclusion criteria (12 patients with acute HF, 147 individuals with HFrEF and HFmrEF, 19 patients with acute myocardial infarction or unstable angina, 9 patients with acute infections, 7 individuals with malignancy, 2 patients with cognitive dysfunction, 72 patients with severe comorbidities, 5 patients with T1DM, 1 patients with gestational diabetes, 26 patients due to surgery list).

We pre-screened 1253 adult patients with HFpEF in our local database according to the conventional criteria: left ventricular (LV) ejection fraction (EF) >50%; abnormal LV global longitudinal strain (GLS) < −16%, LV myocardial mass index (MMI) ≥ 95 g/m^2^ in women or ≥115 g/m^2^ in men, left atrial volume index (LAVI) > 34 mL/m^2^, and abnormal diastolic index E/e’ ≥ 13 units, N-terminal natriuretic pro-peptide (NT-proBNP) ≥ 300 pmol/mL [41]. Finally, between October 2020 and August 2022 were prospectively enrolled 953 outpatients with hemodynamically stable HFpEF and a presence of sinus rhythm on ECG and without a previous history of AF in the following medical centers: the private hospital Vita-Centre (Zaporozhye, Ukraine), EliteMedService (Zaporozhye, Ukraine), City Hospital #7 (Zaporozhye, Ukraine) and the private hospital “MIRUM clinic” (Kyiv, Ukraine). All patients continued to receive optimal GDMT and provided voluntary written informed consent to participate in the study (Figure 1). Major exclusion criteria were acute HF, HFrEF, HF with mildly reduced ejection fraction (HFmrEF), acute myocardial infarction, unstable angina, recent stroke/transient ischemic attack, acute viral and bacterial infection, known malignancy, active chemotherapy, with severe comorbidities, including end stage renal disease (ESRD), cognitive dysfunction/dementia, pregnancy/gestation. Patients were followed for 3 years and divided into two cohorts based on the development of AF: 172 patients exhibited AF, whereas 781 individuals preserved sinus rhythm on ECG.

### 2.2. Determination of AF and Follow-Up

We determined 3-year cases of any forms of AF according to 2020 ESC Guidelines for the diagnosis and management of AF [42]. To detect AF we utilized ECG at rest, continuous 72-h ECG monitoring (when needed), direct interview with patients and/or their relatives, contact with general practitioners, review of databases, and discharge reports. The following had to be present for a case of AF to be verified: an absence of distinct repeating P waves on an ECG; irregular atrial activations; and irregular R-R intervals. The patient-centered management of AF involved individualizing treatment strategies according to the conventional approach [43]. CHA2DS2-VASc and HAS-BLED scores were utilized to assess the risk of thromboembolism and bleeding and provide anticoagulation in patients with HFpEF after AF verification. Follow-up data were collected via clinic visits at baseline and during 3 years after the baseline with 3-month intervals.

### 2.3. Echocardiography Examination

In the study, all patients underwent a routine transthoracic B-mode and Doppler ultrasound examination. This was provided by an experienced echocardiographer using a GE Healthcare Vivid E95 scanner in apical 2- and 4-chamber views (General Electric Company, Horton, Norway). The conventional hemodynamic parameters included LVEF by using Simpson’s method, the LV end-diastolic (LVEDV) and end-systolic (LVESV) volumes, the left atrial volume index (LAVI), early diastolic blood filling (E), and the mean longitudinal strain ratio (e‘) were evaluated according to 2018 Guideline of the American Society of Echocardiography [44]. The estimated E/e’ ratio was expressed as the ratio of the E-wave velocity to the average of the medial and lateral e’ velocities. After acquisition of high-quality echocardiographic data during at least three cardiac cycles, LV GLS was obtained by 2D speckle-tracking image analysis. We stored the data in the DICOM format for subsequent analysis. Left ventricular hypertrophy was defined as an LVMMI of ≥95 g/m^2^ in women and ≥115 g/m^2^ in men [44].

### 2.4. Blood Sampling

Blood samples were obtained from all participants in fasting condition and collected in BD Vacutainer Serum Plus Tube. The samples were stored for 30 min at room temperature to clot. After clotting, the samples were centrifuged at 3000 rpm for 15 min at room temperature. Samples with hemolysis were not used for further evaluation. The supernatant was collected and stored at −70 °C until analysis at certified laboratory of the Vita Centre (Zaporozhye, Ukraine).

### 2.5. Biomarkers Assessment

The local Vita Centre laboratory in Zaporozhye, Ukraine, used a Roche P800 analyzer from Basel, Switzerland, to determine conventional hematological and biochemical parameters. The following data were recorded: blood routine indices, electrolytes, liver enzymes, glucose, serum uric acid, serum creatinine and lipid profile (total cholesterol, triglycerides, high-density lipoprotein [HDL-C] and low-density lipoprotein [LDL-C] cholesterol). Concentrations of circulating biomarkers (N-terminal natriuretic pro-peptide [NT-proBNP], high-sensitivity cardiac troponin T [hs-TrT], soluble suppression of tumorigenicity-2 [sST2], galectin-3, tumor necrosis factor-alpha [TNF-α], high-sensitivity C-reactive protein [hs-CRP], and IL-6) were measured in serum using ELISA kits (Elabscience, Houston, TX, USA) in accordance with the manufacturer’s instructions. Adropin levels were measured with ELISA kit produced by Antibodies.com (Stockholm, Sweden). The data obtained from the ELISA analysis were subjected to standard curve-based evaluation. Each sample was analyzed in duplicate and the mean value was used for the final analysis. The intra- and inter-assay coefficients of variation for each marker were both below 10%.

### 2.6. Glomerular Filtration Rate Estimation

The CKD-EPI formula was utilized to estimate the glomerular filtration rate (eGFR) [45].

### 2.7. Determination of Insulin Resistance

The Homeostatic Assessment Model of Insulin Resistance (HOMA-IR) was performed to assess insulin resistance [46].

### 2.8. Statistics

All statistical analyses were conducted using SPSS 11.0 for Windows and Graph Pad Prism, version 9 (Graph Pad Software, San Diego, CA, USA). The data were tested for normality and homogeneity of variance using the Kolmogorov-Smirnov test. All continues variables were expressed as mean ± standard deviation [SD] median and interquartile range [IQR] depending on whether the data were normally distributed, whereas categorical variables were presented as number (n) and percentage (%). Spearman’s correlation coefficient (r) was utilized for correlations between hemodynamic parameters, comorbidities and biomarkers including the levels of adropin. We utilised the Benjamini-Hochberg procedure to calculate the false discovery rate-adjusted *p*-value for each pair of variables. The chi-squared test was used to compare categorical variables. To compare the data between the two groups, an independent samples *t*-test was used if the variances were homogeneous and a Mann–Whitney U test was used if they were not. Receiver operating characteristic (ROC) curves were used to calculate the cutoff values for variables in predicting AF. Youden’s index (sensitivity + specificity − 1) was used to determine the optimal cut-off value for each possible predictor. Univariate and multivariate Cox proportional hazard models were employed to investigate the prognostic factors for AF. The odds ratio (OR) and 95% confidence interval (CI) were reported for each predictor variable. We compared the incremental prognostic capacity of models on using a binary prediction methodology based on the estimation of integrated discrimination indices (IDI) and net reclassification improvement (NRI). A *p*-value of less than 0.05 was considered to suggest a statistically significant difference.

## 3. Results

### 3.1. Baseline Clinical Characteristics

A total of 953 patients with HFpEF, New York Heart Association class II (n = 385)/III (n = 568) and were enrolled in the study and divided into two cohorts depending on a presence of new onset AF (n = 172) or sinus rhythm on ECG (n = 781) during 3 years. The clinical characteristics of patients outlined in Table 1.

The entire group of patients had a mean age of 69 years and 52.2% of them were male. The profile of comorbidity conditions included dyslipidemia (62.2%), hypertension (87.9%), stable coronary artery disease (36.0%), smoking (35.2%), abdominal obesity (29.2%), type 2 diabetes mellitus (37.4%), left ventricular hypertrophy (70.9%), and chronic kidney disease 1–3 stages (26.3%). Therefore, all patients were hemodynamically stable, had a mean systolic/diastolic blood pressure of 143/87 mm Hg, a mean LVEF of 54%, an average of LAVI of 38 mL/m^2^, a mean of GLS of −15.9%. The therapy of HFpEF were personally optimized and included antagonists of renin-angiotensin-aldosterone system (ACE inhibitors, or angiotensin-II receptor blockers or angiotensin receptor-neprilysin inhibitors), beta-blockers, diuretics, sodium–glucose cotransporter-2 inhibitor and other concomitant medications depending on the presence of comorbidities and diseases.

Patients in AF cohort were older, more likely to have type 2 diabetes mellitus and CKD stages 1–3, higher LAVI, concentrations of hs-CRP, NT-proBNP, sST2 and lower adropin levels than those with sinus rhythm on ECG. The patients with HFpEF from patients with sinus rhythm on ECG were more frequently treated with angiotensin-II receptor blockers, beta-blockers, and SGLT2 inhibitors when compared with those who had AF.

We did not find significant differences between the two patient cohorts with respect to gender, body mass index, anthropometric parameters, the presence of dyslipidemia, hypertension, stable coronary artery disease, smoking, abdominal obesity, left ventricular hypertrophy, New York Heart Association classes, systolic and diastolic blood pressure, left ventricular end-diastolic and end-systolic volumes, LVEF, LVMMI, E/e`, GLS, eGFR, lipid profile, glucose levels, HOMA-IR, HbA1c, and concentrations of TNF-alpha, galectin-3, IL-6, and hs-TrT.

### 3.2. Spearman’s Correlations Between Hemodynamic Parameters, Comorbidities and Biomarkers

**Figure 2:**
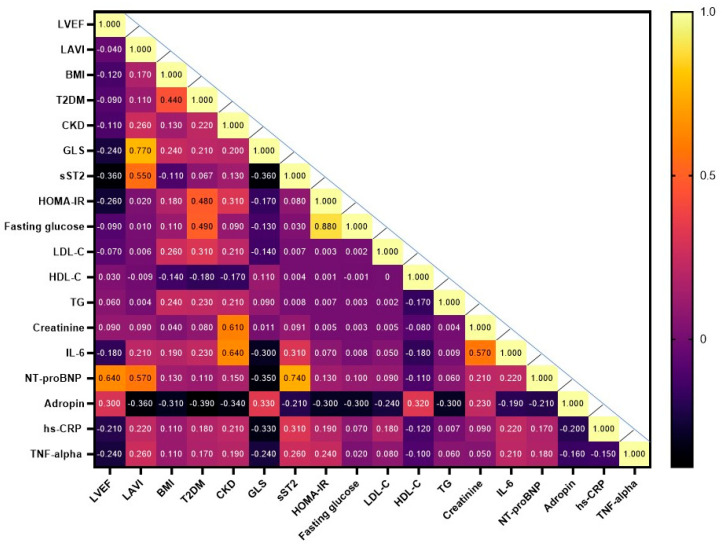
Heat map with Spearmen correlations between each pair of variables. Abbreviations: BMI, body mass index; CKD, chronic kidney disease; hs-CRP, high-sensitivity C-reactive protein; GLS, global longitudinal strain; HOMA-IR, Homeostatic Assessment Model of Insulin Resistance; HDL-C, high-density lipoprotein cholesterol; LAVI, left atrial volume index; LVEF, left ventricular ejection fraction; LDL-C, low-density lipoprotein cholesterol; IL, interleukin; NT-proBNP, N-terminal brain natriuretic pro-peptide; TG, triglycerides; sST2, soluble suppressor tumorigenisity-2; TNF-alpha, tumor necrosis factor-alpha. Note: serum levels of adropin were found to be positively correlated with LV systolic function (LVEF, GLS), kidney function (creatinine) and inversely correlated with left atrial overload, CKD, T2DM, BMI, metabolic parameters (triglyceride, fasting glucose, HOMA-IR, LDL-C) and biomechanical stress biomarker (NT-proBNP).

The heat map provides a visual representation of the Spearman correlation coefficients between each pair of variables including age, gender, hemodynamic parameters, comorbidities and circulating biomarkers (Figure 2). The study has shown that the levels of serum adropin positively correlated with LVEF (r = 0.33; *p* = 0.001), GLS (r = 0.33; *p* = 0.001), HDL-C levels (r = 0.30; *p* = 0.001), creatinine (r = 0.26; *p* = 0.002) and inversely correlated with LAVI (r = −0.36; *p* = 0.001), CKD (r = −0.34; *p* = 0.001), T2DM (r = −0.39; *p* = 0.001), BMI (r = −0.31; *p* = 0.001), triglyceride levels (r = −0.30; *p* = 0.001), fasting glucose (r = −0.30; *p* = 0.001), HOMA-IR (r = −0.30; *p* = 0.012), LDL-C (r = −0.24; *p* = 0.001) and NT-proBNP (r = −0.21; *p* = 0.012).

### 3.3. Predictive Factors of AF: The Results of the Receiver Operating Characteristic Curve Analysis

ROC curve analysis was performed to determine the optimal cutoff for possible predictors of new onset AF in patients with HFpEF (Table 2). Age ≥ 75 years showed the area under curve (AUC) for AF of 0.697 (95% confidence interval [CI] = 0.712–0.740, *p* = 0.001) with a sensitivity of 70.3% and a specificity of 61.6%.

**Figure 3:**
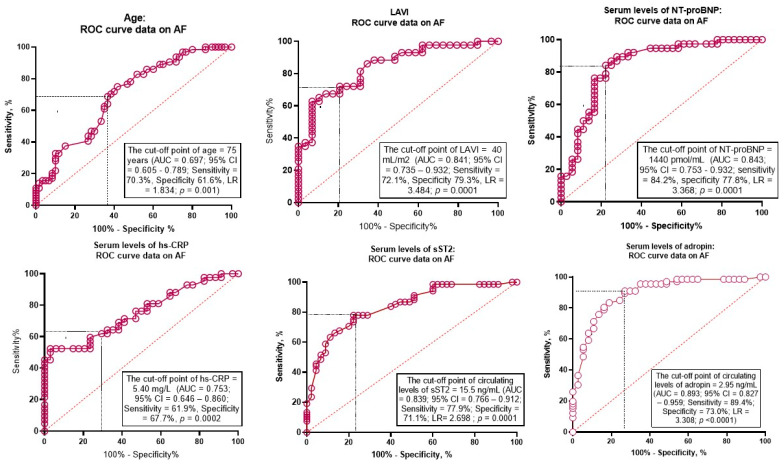
Receiver Operating Characteristic Curves for possible predictors of AF. Abbreviations: AUC, area under curve; CI, confidence interval; ROC, Receiver Operating Characteristic Curve; LR, positive likelihood ratio; LAVI, left atrial volume index; hs-CRP, high-sensitivity C-reactive protein; sST2, soluble suppression tumorigenisity-2; NT-proBNP, N-terminal brain natriuretic pro-peptide.

The well-balanced predictive cutoff point for LAVI was 40 mL/m^2^ (AUC = 0.841, 95% CI = 0.735–0.932) with a sensitivity of 72.1% and a specificity of 79.3%. The AUC for NT-proBNP was 0.843 (95% CI = 0.751–0.932) and predictive cutoff point was 1440 pmol/mL (sensitivity = 84.2%; specificity = 77.8%). Optimal cutoff points for hs-CRP and sST2 were 5.4 mg/L (AUC = 0.753, 95% CI = 0.646–0.860, sensitivity = 61.9%; specificity = 67.7%) and 15.5 ng/mL (AUC = 0.839, 95% CI = 0.766–0.912; sensitivity = 77.9%; specificity = 71.1%), respectively. The AUC for adropin was 0.893 (95% CI = 0.827–0.959) and predictive cutoff point was 2.95 ng/mL (sensitivity = 89.4%; specificity = 73.0%). ROC curves for these variables are shown in Figure 3.

### 3.4. Predictive Factors for New Onset AF: Unadjusted and Adjusted for Multivariate Cox Proportional Hazard Models

Cox proportional hazards model was constructed for assessment of independent predictors of AF (Table 3). Significant variables (*p* < 0.05) comparing cohorts with and without AF were entered into univariate Cox regression analysis, and those retaining statistical significance (*p* < 0.05) were entered into multivariate Cox proportional hazards model.

In unadjusted rough Cox regression model, age ≥ 75 years, type 2 diabetes mellitus, CKD stages 1–3, LAVI ≥ 40 mL/m^2^, NT-proBNP ≥ 1440 pmol/mL, hs-CRP ≥ 5.40 mg/L, adropin ≤ 2.95 ng/mL, sST2 ≥ 15.5 ng/mL were identified as the predictors for new onset AF in HFpEF patients. After adjusting for age ≥ 75 years (Model 2), the presence of type 2 diabetes mellitus, CKD stages 1–3, LAVI ≥40 mL/m^2^, NT-proBNP ≥ 1440 pmol/mL, adropin ≤ 2.95 ng/mL had discriminative values for AF. Model 3 has revealed that the only the levels of NT-proBNP ≥ 1440 pmol/mL and adropin ≤ 2.95 ng/mL were independent predictors of AF.

### 3.5. Subgroup Analyses by Sex and BMI

To further confirm the association between the levels of adropin and the risk of AF, subgroup analyses stratified with sex (male vs female), BMI (<29 kg/m^2^ vs. ≥30 kg/m^2^), T2DM and CKD stages 1–3 were performed (Table 4). The analysis did not define significant interactions between subgroups including sex and BMI (*p* for interaction = 0.166 and *p* for interaction = 0.122, respectively), whereas comparisons of patients with T2DM versus non-T2DM patients and individuals with CKD 1–3 stages versus non-CKD 1–3 stages revealed significant interactions (*p* for interaction < 0.001 for all cases).

### 3.6. Comparison of the Predictive Models

We found that discriminative value of adropin was superior to NT-proBNP, while adding adropin to NT-proBNP did not improve predictive information of adropin alone (Table 5).

### 3.7. Reproducibility of Adropin Versus NT-proBNP

The assessment of the reproducibility regarding adropin compared with NT-proBNP has shown that the intra-class correlation coefficients for inter-observer reproducibility of NT-proBNP and adropin were 0.75 (95% CI = 0.69–0.82) and 0.86 (95% CI = 0.80–0.91), respectively. We did not find significant changes in intra-observer reproducibility of adropin in relation to age and gender in eligible patients (*p* = 0.316 and *p* = 0.412).

## 4. Discussion

In the 3-year longitudinal multicenter study, we found that age, type 2 diabetes mellitus, CKD stages 1–3, increased LAVI (≥40 mL/m^2^), elevated levels of NT-proBNP (≥1440 pmol/mL), hs-CRP (≥5.40 mg/L), sST2 (≥15.5 ng/mL) and low levels of adropin (≤2.95 ng/mL) significantly predicted incident AF. After adjusting for age, the presence of type 2 diabetes mellitus and early stages of CKD, elevated levels of NT-proBNP, adropin remained independent predictors for AF in this patient population. Moreover, we have first identified a higher predictive value of low circulating adropin when compared with elevated NT-proBNP for new episodes of AF in symptomatic patients with HFpEF.

Despite a wide implementation of international guideline for AF in the routine clinical praxis, identifying patients at high risk of new AF remains challenging. Previous clinical studies and meta-analysis have shown that AF episodes and their progression from paroxysmal to non-paroxysmal forms depend on combinations of several conditions and parameters. They may include older age, a history of angina pectoris, myocardial infarction, hypertension, diabetes, thyroid dysfunction, LVH, cardiac surgery, cardiomyopathies, LA asynchrony and LA remodeling, heart rhythm disturbance, LV diastolic and systolic dysfunction, low hematocrit and hemoglobin, elevated levels of HbA1c, natriuretic peptides, galectin-3, sST2, and hs-CRP [47,48,49,50,51]. There is little evidence for HFpEF patients without a previous history of AF who were treated with optimal GDMT. Although elevated level of NT-proBNP was a predictor of incident AF during the first 2 years of long-term observation, it remains unclear, whether the discriminative value of natriuretic peptide(s) remains accurate in individuals with HFpEF and coexisting overweight, obesity or type 2 diabetes mellitus [52,53]. Indeed, glycosylation and obesity may affect the prognostic value of NT-proBNP regarding AF [54,55], while NT-pro-BNP significantly improved the predictive ability of conventional cardiovascular risk factors and the novel CHARGE-AF risk score for AF [56]. On the other hand, the biomarkers, which relate to cardiac fibrosis (galectin-3, sST2), inflammation (ha-CRP, IL-6, TNF-alpha) and myocardial injury (cardiac troponins), did not provide the superiority over NT-proBNP in predicting AF in individuals without previous incident of AF [56,57]. In this context, a discovery of new non-invasive, easily accessible biomarkers that could stratify HFpEF patients depending on the risk of new onset AF appears to be promising.

In our study, we have identified low levels of adropin as promising biomarker of higher risk of AF in HFpEF patients who were optimally treated with GDMT and had target/near target levels of NT-proBNP (<1000 pmol/mL). These findings are likely to be practically useful, because they allow providing a clear approach to risk stratification beyond respectively time-consuming analysis of LA remodeling. Because in recent clinical studies has been proven a linear correlation between serum adropin concentrations and LA diameter, LAVI, LA strain in patients with AF [58,59], it could suggest that a decrease in the concentration of this peptide may precede the manifestation of AF and LA volume overload.

Adropin is likely to be a promising prognostic biomarker for adverse cardiac remodeling and HF. Unfortunately, there is little rigorous clinical evidence of the superiority of adropin over natriuretic peptides and other biomarkers of biomechanical stress, inflammation, cardiac fibrosis, and myocardial damage. Most of them were obtained in cohort studies and require further validation [32,34,37,39,59]. Nevertheless, our findings suggest that adropin may play a protective role in cardiovascular remodeling and that its deficiency represents a metabolic maladaptation that potentiates accelerated HF progression. Indeed, extensive researches have explored the pivotal role of adropin, particularly in mechanisms related to inflammation, immune response and oxidative stress through suppression of VEGFR2/phosphatidylinositol 3-kinase/AKT and VEGFR/c-Src/ERK1/2 signaling [60]. Moreover, adropin demonstrated direct cardiac and vascular protective effects via modulating brain-derived neurotrophic factor and inhibiting SIRT1 and TNF-alpha-depending apoptosis [61].

Another aspect of an association of low levels of adropin with the risk of AF is their possible link with endothelial dysfunction, accelerating atherosclerosis and lipid toxicity. Perhaps the loss of the suppressive effect of adropin on ROR-dependent regulation of lipid oxidation may significantly mediate its negative effect on the lipid core formation in atherosclerotic plaques, sub-intimal accumulation of oxidized lipoproteins, disruption of integral functions of the vascular wall, and regulation of vasodilation [60,62]. Therefore, adropin downregulated lipogenic proteins SEBP-1 and ADRP and thereby potentiates lipid toxicity, ROS production and mitochondrial dysfunction [63]. Overall, all these pathogenetic pathways are likely to be crucial for AF development.

To note, in our study we did not find any significant differences in glucose homeostasis parameters, lipid profile between HFpEF patients with AF and those with sinus rhythms, whereas adropin levels were sufficient different. However, the associations between adropin levels and the parameters mentioned above were found. We hypothesized that adropin playing an important role in the overall regulation of glucose and lipid metabolism is not the plausible factor that mediates variability of these parameters. On the contrary, liver perfusion and hepatocyte metabolism are likely to be in the loop of the regulation of adropin levels in circulating blood. Along with it, adipose tissue inflammation and concomitant diabetes may intervene in expression of adropin in remote tissue and thereby limit organ protective capability of the peptide.

Finally, as we had hypothesized, adropin is a promising indicator of a higher risk of new onset AF in individuals with HFpEF, whose predictive potency exceeds that of NT-proBNP. These findings open up a new perspective for a large-scale clinical study to develop a new risk stratification model for these patients.

## 5. Study Limitations

The study has several limitations. The first limitation affects the lack of data regarding a trajectory of biological markers during the observational period in connection with new cases of AF regardless of their form. Additionally, we did not investigate nutritional status among the patients and its association with the changes of adropin levels. Therefore, a three-year observation period is probably not long enough for an evaluation of all episodes AF. The clinical applicability of adropin testing is currently the subject of scientific discussion, as there are still limited clear indications for its use in routine clinical practice. Nevertheless, there are now affordable ELISA kits available that have the potential to be standardized in clinical studies. Additionally, validation of the adropin cut-off in an external cohort has not been provided. The next limitation affects the geographical scope of the study (Ukraine), which has the potential to restrict external validity. Finally, we did not compare the discriminative value of adropin with previously validate risk scores, such as Cohorts for Heart and Aging Research in Genomic Epidemiology model for atrial fibrillation (CHARGE-AF). However, we do not believe that these limitations will have an impact on the interpretation of the results.

## 6. Conclusions

We found that after adjusting for age ≥ 75 years, a presence of type 2 diabetes mellitus and CKD stages 1–3, the levels of NT-proBNP ≥ 1440 pmol/mL and adropin ≤ 2.95 ng/mL were independent predictors of new onset AF in patients HFpEF. Moreover, adropin ≤ 2.95 ng/mL presented more predictive information than NT-proBNP ≥ 1440 pmol/mL alone, whereas the combination of both biomarkers did not improve the predictive ability of adropin alone.

## Figures and Tables

**Table 1:** Basic characteristics of the patients involved in this study.

Variables	Entire Group Patients with HFpEF (*n* = 953)	Patients with HFpEF and AF (*n* = 172)	Patients with HFpEF and Sinus Rhythm (*n* = 781)	*p* Value
Demographics and anthropomorphic parameters				
Age (years)	69 (54–85)	75 (61–87)	66 (52–81)	0.042
Male (*n* (%))	497 (52.2)	92 (53.5)	405 (51.9)	0.818
BMI (kg/m^2^)	26.9 ± 7.20	27.6 ± 6.10	25.8 ± 5.80	0.680
Waist circumference (cm)	99 ± 8	101 ± 5	98 ± 9	0.730
WHR (units)	0.90 ± 0.13	0.91 ± 0.10	0.90 ± 0.14	0.850
Medical history				
Dyslipidemia (*n* (%))	588 (62.0)	108 (62.8)	480 (61.5)	0.846
Hypertension (*n* (%))	838 (87.9)	153 (88.9)	685 (87.7)	0.890
Stable CAD (*n* (%))	343 (36.0)	64 (37.2)	279 (35.7)	0.388
Dilated CMP, (*n* (%))	58 (6.1)	12 (7.0)	46 (5.9)	0.482
Smoking (*n* (%))	335 (35.2)	65 (37.7)	270 (34.5)	0.358
Abdominal obesity (*n* (%))	278 (29.2)	48 (27.9)	230 (29.4)	0.642
Type 2 diabetes mellitus, (*n* (%))	356 (37.4)	72 (41.9)	284 (36.4)	0.042
LVH (*n* (%))	676 (70.9)	128 (74.4)	548 (70.2)	0.056
CKD stages 1–3 (*n* (%))	251 (26.3)	69 (40.1)	182 (23.3)	0.043
New York Heart Association class II/III	385 (40.4)/568 (59.6)	65 (37.8)/107 (62.2)	320 (41.0)/461 (59.0)	0.710
Hemodynamics				
Systolic BP (mm Hg)	143 ± 11	147± 8	140 ± 13	0.840
Diastolic BP (mm Hg)	87 ± 9	86 ± 9	87 ± 10	0.780
LVEDV (mL)	158 (144–173)	160 (142–181)	157 (140–178)	0.650
LVESV (mL)	73 (63–82)	73 (62–86)	71 (60–83)	0.612
LVEF (%)	54 (51–57)	53 (51–56)	55 (52–59)	0.545
LVMMI (g/m^2^)	144 ± 16	148 ± 19	142 ± 18	0.477
LAVI (mL/m^2^)	38 (33–44)	40 (35–48)	37 (32–43)	0.046
E/e` (units)	19 ± 6	20 ± 4	19 ± 5	0.811
GLS (%)	−17.9 (−16.4; −19.2)	−18.2 (−16.7; −19.8)	−17.1 (−16.3; −19.5)	0.266
Biomarkers				
Hemoglobin, g/L	146 (132–158)	144 (131–155)	147 (130–162)	0.673
eGFR (mL/min/1.73 m^2^)	81 ± 17	77 ± 19	83 ± 15	0.533
Fasting glucose (mmol/L)	4.95 ± 0.9	5.07 ± 1.1	4.91 ± 1.2	0.810
HOMA-IR (units)	7.43 ± 2.5	7.47± 2.3	7.38 ± 2.8	0.650
HbA1c (%)	6.42 ± 0.16	6.45 ± 0.15	6.38 ± 0.20	0.711
Creatinine (µmol/L)	109.8 ± 21.6	115.6± 20.1	102.2 ± 23.2	0.520
SUA (µmol/L)	347 ± 120	359 ± 118	346 ± 135	0.351
Total cholesterol (mmol/L)	5.72 ± 1.30	5.80 ± 1.25	5.70 ± 1.28	0.433
HDL-C (mmol/L)	0.99 ± 0.15	1.01 ± 0.14	0.99 ± 0.16	0.355
LDL-C (mmol/L)	3.84± 0.22	3.90 ± 0.20	3.81± 0.22	0.590
Triglycerides (mmol/L)	2.21 ± 0.16	2.23 ± 0.15	2.20 ± 0.17	0.620
hs-CRP (mg/L)	6.05 (2.98–9.17)	6.38 (3.18–9.75)	5.87 (2.24–9.56)	0.048
TNF-alpha (pg/mL)	3.46 (2.15–4.86)	3.52 (2.09–4.93)	3.29 (1.98–4.80)	0.580
NT-proBNP (pmol/mL)	1068 (375–1606)	1360 (532–1850)	986 (343–1657)	0.042
Adropin (ng/mL)	3.48 (1.58–5.45)	2.93 (1.25–4.58)	3.72 (1.69–5.80)	0.044
Galectin-3 (ng/mL)	4.24 (1.15–7.46)	4.31 (1.08–7.55)	4.15 (1.01–7.31)	0.312
IL-6 (pg/mL)	1.98 (0.80–3.15)	2.14 (0.93–3.21)	1.92 (0.77–3.09)	0.060
sST2 (ng/mL)	11.2 (3.46–18.9)	14.3 (3.54–20.2)	10.9 (2.87–19.1)	0.044
hs-TnT, ng/mL	0.06 (0.011–0.117)	0.07 (0.013–0.122)	0.05 (0.005–0.116)	0.548
Concomitant medications				
ACEIs (*n* (%))	715 (75.0)	123 (71.5)	592 (75.8)	0.174
ARBs (*n* (%))	152 (15.9)	24 (14.0)	128 (16.4)	0.046
ARNI, (*n* (%))	36 (3.8)	5 (2.9)	31 (4.0)	0.050
Beta-blockers (*n* (%))	851 (89.3)	137 (79.7)	714 (91.4)	0.048
Ivabradine (*n* (%))	137 (14.4)	18 (10.5)	119 (15.2)	0.046
CCBs (*n* (%))	181 (19.0)	33 (19.2)	148 (19.0)	0.880
Loop and thiazide-like diuretics (*n* (%))	891 (93.5)	163 (94.8)	728 (93.2)	0.835
Antiplatelet agents (*n* (%))	793 (83.2)	144 (83.7)	649 (83.1)	0.882
Anticoagulants (*n* (%))	91 (9.5)	15 (8.7)	76 (9.7)	0.828
Metformin (*n* (%))	325 (34.1)	57 (33.1)	268 (34.3)	0.850
DPP-4 inhibitors (*n* (%))	31 (3.2)	5 (2.9)	26 (3.3)	0.162
GLP-1 RAs (*n* (%))	48 (5.0)	8 (4.7)	40 (5.1)	0.116
SGLT2 inhibitors (*n* (%))	811 (85.0)	127 (73.8)	684 (87.6)	0.042
Statins (*n* (%))	856 (89.8)	152 (88.4)	704 (90.1)	0.820

Notes: Variables are given as Ms ± SDs or Ms (25–75% IQRs). The Chi-square test was used to compare categorical variables. The Mann–Whitney U test, and Chi-square test were used to compare continuous variables between cohorts. Abbreviations: ARBs, angiotensin-II receptor blockers; ACEIs, angiotensin converting enzyme inhibitors; ARNI, angiotensin receptor-neprilysin inhibitors; BMI, body mass index; CAD, coronary artery disease; CMP, cardiomyopathy; CKD, chronic kidney disease; CCBs, calcium channel blockers; DPP-4, dipeptidyl peptidase-4; eGFR, estimated glomerular filtration rate; E/e‘, early diastolic blood filling to longitudinal strain ratio; GLS, global longitudinal strain; GLP-1 RAs, glucagon-like peptide-1 receptor agonists; HDL-C, high-density lipoprotein cholesterol; hs-CRP, high-sensitivity C-reactive protein; hs-TnT, high-sensitivity troponin T; LAVI, left atrial volume index; LDL-C, low-density lipoprotein cholesterol; LVH, left ventricular hypertrophy; LVEDV, left ventricular end-diastolic volume; LVESV, left ventricular end-systolic volume; LVEF, left ventricular ejection fraction; LVMMI, left ventricle myocardial mass index; IL, interleukin; NT-proBNP, N-terminal natriuretic pro-peptide; SGLT2, sodium–glucose cotransporter-2; SUA, serum uric acid; sST2, soluble suppressor tumorigenisity-2; TNF-alpha, tumor necrosis factor-alpha; WHR, waist-to-hip ratio.

**Table 2:** Receiver Operating Characteristic (ROC) Curve Analysis for Predictive Factors of AF.

Variables	AUC	95% CI	*p* Value	Cutoff	Se, %	Sp, %
Age	0.697	0.605–0.789	0.0002	75 years	70.3	61.6
LAVI	0.841	0.735–0.932	0.0001	40 mL/m^2^	72.1	79.3
NT-proBNP	0.843	0.751–0.932	0.0001	1440 pmol/mL	84.2	77.8
hs-CRP	0.753	0.646–0.860	0.0002	5.40 mg/L	61.9	67.7
Adropin	0.893	0.827–0.959	0.0001	2.95 ng/mL	89.4	73.0
sST2	0.839	0.766–0.912	0.0001	15.5 ng/mL	77.9	71.1

Abbreviations: AUC, area under curve; CI, confidence interval; LAVI, left atrial volume index; Se, sensitivity; Sp, specificity; hs-CRP, high-sensitivity C-reactive protein; sST2, soluble suppressor tumorigenisity-2; NT-proBNP, N-terminal brain natriuretic pro-peptide.

**Table 3:** Cox regression analysis for predictive factors of AF.

Predictive Factors	Model 1		Model 2		Model 3	
	HR (95% CI)	*p* Value	HR (95% CI)	*p* Value	HR (95% CI)	*p* Value
Age ≥ 75 years	1.215 (1.046–1.448)	0.046	-		-	
LVH (presence vs. absent)	1.036 (0.988–1.122)	0.466	-		-	
T2DM (presence vs. absent)	1.366 (1.124–1.578)	0.044	1.325 (1.118–1.543)	0.048	-	
CKD stages 1–3 (presence vs. absent)	1.292 (1.164–1.459)	0.012	1.203 (1.018–1.422)	0.050	-	
New York Heart Association HF class (III vs. II)	1.216 (1.002–1.452)	0.064	-		-	
LAVI ≥ 40 mL/m^2^	1.533 (1.117–1.956)	0.001	1.411 (1.104–1.847)	0.026	-	
NT-proBNP ≥ 1440 pmol/mL	1.497 (1.125–2.833)	0.001	1.541 (1.116–2.253)	0.001	1.536 (1.120–2.247)	0.001
hs-CRP ≥ 5.40 mg/L	1.126 (1.014–1.843)	0.048	1.088 (1.035–1.106)	0.052	1.049 (1.013–1.098)	0.144
Adropin ≤ 2.95 ng/mL	1.783 (1.255–2.815)	0.001	1.696 (1.247–2.990)	0.001	1.690 (1.240–2.864)	0.001
sST2 ≥ 15.5 ng/mL	1.246 (1.112–1.878)	0.012	1.215 (1.088–1.830)	0.051	1.176 (1.043–1.820)	0.062

Notes: Model 1: unadjusted rough model; Model 2: model adjusted for age ≥ 75 years; Model 3: model adjusted for age ≥ 75 years and the presence of concomitant conditions (type 2 diabetes mellitus, CKD stages 1–3) and LAVI. Abbreviations: AF, atrial fibrillation; CI, confidence interval; DM, diabetes mellitus; LAVI, left atrial volume index; LVH, left ventricular hypertrophy; HF, heart failure; HR, hazard ratio; hs-CRP, high-sensitivity C-reactive protein; sST2, soluble suppressor tumorigenisity-2; NT-proBNP, N-terminal brain natriuretic pro-peptide.

**Table 4:** Subgroup analyses stratified by sex, BMI, T2DM and CKD stages 1–3.

Subgroup	Event Rates (Number of Patients with AF/Entire Patients, %)	OR (95% CI) per 0.25 ng/mL Decrease of Adropin	*p* for Interaction
Entire group	172/953, 18.0	1.140 (1.045–1.279)	<0.001
Sex			
Male	92/497, 18.5	1.215 (1.012–1.293)	0.166
Female	80/456, 17.5	1.061 (0.961–1.116)	
BMI			
<29 kg/m^2^	124/675, 18.4	1.126 (0.998–1.255)	0.122
≥30 kg/m^2^	48/278, 17.3	1.167 (1.041–1.198)	
T2DM			
no	100/597, 16.8	1.049 (1.026–1.087)	<0.001
yes	72/356, 20.2	1.227 (1.132–1.327)	
CKD stages 1–3			
no	103/702, 14.7	1.082 (1.034–1.129)	<0.001
yes	69/251, 27.5	1.230 (1.121–1.355)	

Notes: ORs were calculated per 0.25 ng/mL decrease of adropin levels. Abbreviations: OR, odds ratio; CI, confidence interval; BMI, body mass index; CKD, chronic kidney disease.

**Table 5:** The comparisons of predictive models for AF.

Models	AUC		NRI		IDI	
	M (95% CI)	*p* Value	M (95% CI)	*p* Value	M (95% CI)	*p* Value
NT-proBNP ≥ 1440 pmol/mL	0.843 (0.751–0.932)	-	Reference		Reference	
Adropin ≤ 2.95 ng/mL	0.893 (0.827–0.959)	0.040	0.25 (0.21–0.30)	0.046	0.41 (0.32–0.52)	0.044
NT-proBNP ≥ 1440 pmol/mL + Adropin ≤ 2.95 ng/mL	0.929 (0.837–0.974)	0.048	0.29 (0.24–0.35)	0.040	0.48 (0.40–0.56)	0.042

Abbreviations: AUC, area under curve; CI, confidence interval; IDI, integrated discrimination indices; M, mean value; NRI, net reclassification improvement; NT-proBNP, N-terminal brain natriuretic pro-peptide.

## Data Availability

The data presented in this study are available on request from the corresponding author due to privacy restrictions.
